# In middle-aged and old obese patients, training intervention reduces leptin level: A meta-analysis

**DOI:** 10.1371/journal.pone.0182801

**Published:** 2017-08-15

**Authors:** Ildikó Rostás, László Pótó, Péter Mátrai, Péter Hegyi, Judit Tenk, András Garami, Anita Illés, Margit Solymár, Erika Pétervári, Ákos Szűcs, Andrea Párniczky, Dániel Pécsi, Zoltán Rumbus, Csaba Zsiborás, Nóra Füredi, Márta Balaskó

**Affiliations:** 1 Institute for Translational Medicine, Medical School, University of Pécs, Pécs, Hungary; 2 Institute of Bioanalysis, Medical School, University of Pécs, Pécs, Hungary; 3 Hungarian Academy of Sciences - University of Szeged, Momentum Gastroenterology Multidisciplinary Research Group, Szeged, Hungary; 4 Division of Gastroenterology, First Department of Internal Medicine, University of Pécs, Pécs, Hungary; 5 First Department of Surgery, Semmelweis University, Budapest, Hungary; 6 Heim Pál Children’s Hospital, Budapest, Hungary; Vanderbilt University, UNITED STATES

## Abstract

**Background:**

Leptin is one of the major adipokines in obesity that indicates the severity of fat accumulation. It is also an important etiological factor of consequent cardiometabolic and autoimmune disorders. Aging has been demonstrated to aggravate obesity and to induce leptin resistance and hyperleptinemia. Hyperleptinemia, on the other hand, may promote the development of age-related abnormalities. While major weight loss has been demonstrated to ameliorate hyperleptinemia, obese people show a poor tendency to achieve lasting success in this field. The question arises whether training intervention *per se* is able to reduce the level of this adipokine.

**Objectives:**

We aimed to review the literature on the effects of training intervention on peripheral leptin level in obesity during aging, in order to evaluate the independent efficacy of this method. In the studies that were included in our analysis, changes of adiponectin levels (when present) were also evaluated.

**Data sources:**

3481 records were identified through searching of PubMed, Embase and Cochrane Library Database. Altogether 19 articles were suitable for analyses.

**Study eligibility criteria:**

Empirical research papers were eligible provided that they reported data of middle-aged or older (above 45 years of age) overweight or obese (body mass index above 25) individuals and included physical training intervention or at least fitness status of groups together with corresponding blood leptin values.

**Statistical methods:**

We used random effect models in each of the meta-analyses calculating with the DerSimonian and Laird weighting methods. I-squared indicator and Q test were performed to assess heterogeneity. To assess publication bias Egger’s test was applied. In case of significant publication bias, the Duval and Tweedie's trim and fill algorithm was used.

**Results:**

Training intervention leads to a decrease in leptin level of middle-aged or older, overweight or obese male and female groups, even without major weight loss, indicated by unchanged serum adiponectin levels. Resistance training appears to be more efficient in reducing blood leptin level than aerobic training alone.

**Conclusions:**

Physical training, especially resistance training successfully reduces hyperleptinemia even without diet or major weight loss.

## Introduction

Obesity is a relentlessly growing public health problem [1, 2] in modern societies due to our increasingly sedentary lifestyle and imbalanced dietary choices. This obesity epidemic increases morbidity and mortality due to consequent cardiometabolic disorders including type 2 diabetes mellitus [3, 4], hypertension and coronary heart disease [5], atherosclerosis and ischemic stroke [6] and also due to certain malignancies [7–9].

The adipose tissue participates in numerous endocrine functions via the secretion of bioactive molecules, such as adipokines [10]. Among adipokines, leptin and adiponectin are the most abundantly secreted peptides [11]. Their role in the regulation of whole body energy metabolism, carbohydrate and lipid metabolism is well-documented [12–17].

Leptin is one of the major adipokines, its increased level indicates the severity of fat accumulation in obesity [12]. Hyperleptinemia is also an important etiological factor of consequent cardiometabolic syndrome and numerous inflammatory disorders [12, 13, 17, 18]. Additionally, its involvement in a number of malignancies has also been suggested by various meta-analyses [19–21].

Aging has been demonstrated to aggravate fat accumulation and to induce leptin resistance and consequent hyperleptinemia [22]. On the other hand, leptin resistance and hyperleptinemia may promote the development of age-associated cardiometabolic abnormalities [23] and autoimmune diseases [18].

Therefore, obesity- and/or age-related hyperleptinemia is not simply an indicator of metabolic abnormalities, but it is also an important factor in the pathogenesis of their complications. As a consquence, interventions aiming to decrease hyperleptinemia may also reduce related morbidity.

While major weight loss has been demonstrated to ameliorate hyperleptinemia, obese people show poor tendency to achieve lasting success in this field [24]. Healthier diets, without significant weight loss, fail to improve hyperleptinemia, although some other inflammatory mediators such as C-reactive protein reacted favorably to them [25–26]. On the other hand, long-term physical (resistance) training without dietary intervention or significant improvement of body composition resulting in only moderate weight loss (< 5%) has been reported to successfully decrease hyperleptinemia along with insulin resistance [7]. In the background, training-induced suppression of the intramyocellular lipid concentrations were assumed [4, 7]. In addition to its well-documented insulin resistance-inducing effect, high intracellular lipid content in muscles has been shown to contribute also to peripheral leptin resistance [4]. In obese individuals with high intramyocellular lipid content, the ability of leptin to repartition fatty acids toward oxidation and away from storage in muscles was suppressed [4]. These leptin effects were restored by physical training that successfully reduced intramyocelllular lipid levels [4, 7].

The question arises whether physical training *per se* [i.e., training without dietary intervention and/or major weight loss) reduces the level of leptin, thereby improving the metabolic status and morbidity rate of obese and aging populations.

We aimed to review the literature on the effects of physical training on peripheral leptin level as indicator of metabolic health in obesity during aging. Our objective was to evaluate the independent efficacy of this intervention on peripheral leptin levels and to suggest new approaches for future clinical studies. In those studies that were included in our analysis, changes of adiponectin levels (when present) were also evaluated.

## Methods

### Search strategy and study selection

We performed this study following principles of the PRISMA statement [27] (S1 Table). No review protocol was registered for this meta-analysis. The search was initially conducted in May 2016, and subsequently updated on April 3rd 2017. Our meta-analysis was based on PICO (P: obese individuals over 45 years of age, I: high physical fitness, C: low physical fitness, O: blood leptin concentration). Records were identified through searching of PubMed (http://www.ncbi.nlm.nih.gov/pubmed), Embase (https://www.embase.com) and Cochrane Library Database (http://www.cochranelibrary.com) Search strategy of PubMed was as follows: (leptin AND ('muscle strength' OR 'muscle power' OR 'muscle force' OR exercise OR training) AND (overweight OR obese OR obesity). This search identified 1207 records. Similar searches were conducted in Embase and Cochrane Library Database. One additional record was identified through references of included studies. Altogether 3481 records were found.

Exclusion criteria included animal experiments, non-English language reports, studies with participants below 45 years of age or any dietary intervention.

Clinical studies [randomized controlled clinical trials (RCT), intervention studies and cohort studies] were eligible provided that they reported data of participants above 45 years of age. [This age cut-off was chosen based on the findings of the National Health Interview Survey, United States, 2003–2009. This study showed that the prevalence of complex activity limitations (sedentary lifestyle) started to rise steeply in the age-group 45–64 years, as compared with that of younger populations (18–44 years) [28]. It continued to increase strongly in older (65–74 years or 75+) age-groups.] Clinical studies had to contain at least one participant group of mean BMI higher than 24.9 (overweight or obese) and they had to report fitness status of groups together with corresponding blood leptin levels. Most studies (18 out of 19) reported effects of physical training intervention (of at least 10 weeks duration or longer) with respective blood leptin levels before and after the intervention.

Due to the restricted availability of training intervention studies that tested peripheral leptin levels of their participants, we have not excluded any of them based on the assessment of their individual risk of bias (lack of randomized design or low participant number). Due to weighting methods, data with low participant numbers were assigned with lower weights during the analysis.

### Data extraction and evaluation procedure

Records were identified from three databases (PubMed, Embase, Cochrane Library). One additional study was added from the reference list of one of the articles. After removal of the duplicates, two authors (I.R. and J.T.) independently screened the titles and abstracts. Any discrepancy about the eligibility of studies was resolved by discussion. Articles based on their inappropriate topics of interest or conference abstracts without data were also removed. Review papers and editorials, reports of animals experiments, clinical studies focusing on inappropriate patient populations (e.g. too young or with severe comorbidity or severe medical condition or receiving special treatments, etc.) or reports with unsuitable study design (e.g. dietary intervention, acute training, etc.) were excluded. The remaining records were assessed based on their full-texts. Those full-text articles not reporting applicable data for statistics, focusing on different patient populations, with inappropriate study design or written in non-English language had to be excluded, as well. We included only those articles that focused on groups over 45 years with BMI above 24.9, free of serious medical conditions or severe comorbidities and reported pre- and post-training blood leptin values appropriate for statistical analysis. The blood leptin values of one article that simply compared data of a healthy sedentary and a healthy physically active group were not included in the forest plots, since no training intervention was performed in that study. These data were used only for the fitness category-based additional analysis.The following pieces of information were recorded for each study: first author, publication year, sample size, gender, age, body mass index (BMI) values, training type, indicator of the fitness status and blood leptin levels. When available, changes in fat mass and pre- and post-training adiponectin levels were also extracted. Indicators of metabolic health concerning carbohydrate metabolism (such as fasting glucose, fasting insulin, HOMA or QUICKI) were also recorded, when available. No study reported dietary data or values of caloric intake. No supplementary information was obtained from investigators of the original clinical studies, only published data were used.

The risk of bias of individual studies was assessed by the Cochrane Collaboration's tool [29] (S2 Table). The quality of the body of evidence was assessed using the Grading of Recommendations Assessment, Development and Evaluation (GRADE) score according to the British Medical Journal (BMJ) Clinical Evidence approach [30] (S3 Table).

### Determination of fitness categories

Different studies described the fitness status of their participant groups in various terms using different parameters. Therefore, we assigned participant groups into 4 fitness categories based on the available description and/or on the provided parameters, such as steps per day, maximal oxygen consumption, training intensity, etc. “Sedentary” or “inactive” groups, as stated by the studies, were assigned to category 4; “low activity” (e.g. 5000 steps per day characterizing otherwise inactive participants or fitness status following an at least 10-12-week low intensity training intervention in previously sedentary participants) corresponded to category 3; “normal activity” (e.g. normal maximal oxygen consumption or following high intensity training intervention in previously sedentary participants) corresponded to category 2; “active lifestyle”, as stated by the studies or following high intensity training intervention in previously “low activity” participants, corresponded to category 1.

### Statistical methods

We have applied random effect models in each of the meta-analyses (due to conceptual reasons, even when heterogeneity was small), which were calculated with the DerSimonian and Laird weighting method. Regarding summary measures (PRISMA, [31]), primary analyses demonstrated standardized differences of means with 95% confidence intervals (95% CI) from studies that contained blood leptin (or adiponectin) data obtained before and after training intervention. Since meta-analysis calculations require results expressed as mean ± standard deviation as input format, and some papers presented their data as median and quartiles, we used the transformation suggested by Wan and coworkers [31]. Some studies provided their leptin and adiponectin values as geometrical means. For this reason, we applied standardized difference of means in our primary analyses.

Regarding leptin, subgroup analyses were conducted for different age-groups (for participant groups with mean age below 65 or above 65 years), for different BMI groups (overweight: 25 ≤ BMI < 30 or mildly obese: 30 ≤ BMI < 35), and for males and females.

Additional analysis shows mean leptin values of subgroups assigned to different fitness categories (see section “Determination of fitness categories” above) with 95% CI intervals (with summarized weighted means). In all analyses [except for the evaluation of heterogeneity (Q-test) or that of asymmetry of data (Egger’s test)] results were considered to be significant, when p < 0.05.

We used the meta-regression model to explore the effect of various types of training (aerobic or resistance) on the known correlation between training-induced changes in fat mass and changes in blood leptin levels. In each case, we tested the whole model (simultaneously hypothesized that all coefficients are zero) and reported the regression coefficients, 95% CI-s, standard errors and z tests.

To assess whether the heterogeneity observed among effect sizes could be attributed to random chance or other factors may play a determining role (different clinical methods or diverse participants), I-squared (I^2^) indicator and Q test were performed. I^2^ statistics represent the percentage of effect size heterogeneity that cannot be explained by random chance but by other factors mentioned above. If the Q test is significant, it implies that the heterogeneity among effect sizes reported in the observed studies is more diverse than it could be explained only by random error. We considered the Q test significant if p < 0.1.

To compare the leptin levels of groups of different fitness categories we used subgroup analysis, p < 0.05 indicating significant difference.

To assess the presence of publication bias we used Egger’s test to detect asymmetry in the funnel plot. A significant test result (p < 0.1) indicates the existence of bias. In case of detecting such a bias, the Duval and Tweedie's trim and fill algorithm was used to assess the contribution of the bias to the effect.

All statistical analyses were performed with Comprehensive Metaanalysis Software (Biostat Inc.) and Stata 11 SE (Stata Corp.).

## Results

### Search results

**Fig 1:**
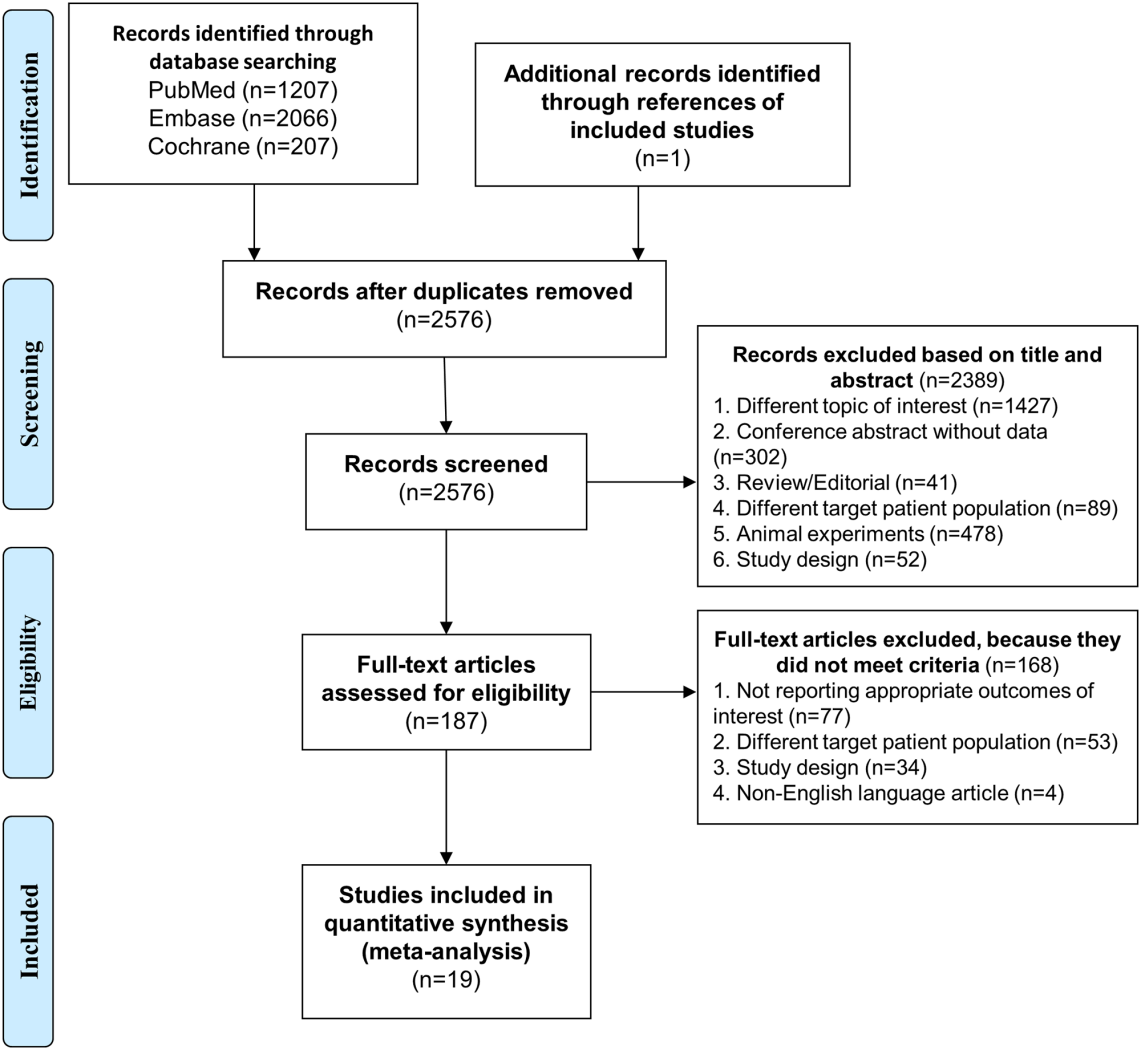
Flowchart of the study selection procedure.

Our database search (up to April 2017) based on PICO (P: obese individuals over 45 years of age, I: high physical fitness, C: low physical fitness, O: blood leptin concentration) identified altogether 3480 records from PubMed (n = 1207), from Embase (n = 2066) and from Cochrane Library database (n = 207). From reference lists of screened studies 1 additional record was obtained. These records were screened for duplicates (905 records) (Fig 1). Further screening was based on title and abstract evaluation of the remaining 2576 records. Following exclusion of articles with inappropriate topics of interest (1427 items), conference abstracts (302 items), review papers and editorials (41 items), reports of animals experiments (478 items), clinical studies with inappropriate patient populations (89 items) or reports with unsuitable study design (52 items), full-texts of the remaining 187 records were assessed. Among them, 77 full-texts failed to report applicable data for statistics, 53 full-texts focused on inappropriate patient populations, 34 full-texts applied inappropriate study design and 4 articles were written in non-English languages. Altogether 19 articles [32–50] proved to be eligible for statistical analysis (Fig 1).

### Study characteristics

Studies used in our meta-analysis dated from 2005 to 2016. Data of 1565 individuals were included in our analysis (number of participants ranged from 10 to 320).

Among the 19 studies (Table 1), 18 applied training intervention [32–45, 47–50], one compared the leptin levels of a mildly obese sedentary and a physically active group with similar BMI [46]. Regarding training, 13 studies applied a single (10-week to 6-month) training period [32, 35, 36, 39, 40–45, 48–50], while 5 studies applied a 2-phase, 12-18-month training with the first leptin test after the first 3 or 6 months [33, 34, 37, 38, 47]. The second phase of training failed to induce further significant decline in leptin levels. Ten studies provided additional pre- and post-training adiponectin levels [32, 33, 36, 38, 40, 41, 44, 45, 48, 49], 13 reported indicators of carbohydrate metabolism (Table 2) [36–45, 48–50], 11 reported training-induced BMI changes [35, 36, 39–45, 49, 50], a partially different subset of 11 studies described training-induced changes in fat mass [34, 39–45, 48–50]. From the 18 studies that investigated the effects of training on leptin levels [32–45, 47–50] only 3 of them failed to detect significant effect [43, 45, 47].

**Table 1:** Description of the studies included in the meta-analyses.

Study	Subgroup	Initial sample size (participants who completed the training)	Age	SD	Sex	BMI	SD	Blood leptin				Correlation between leptin levels and training	Adiponectin	Fat mass
								Before training	SD (or 95% CI)	After training	SD (or 95% CI)			
**Abbenhardt et al. (2013)** [32]	Control group (BMI < 30)	41 (37)	57.4	4.4	F	<30	NR	19.8*	(17.8–22.0)	19.4*	(16.8–22.4)	✓	✓	NR
	Control group (BMI > 30)	46 (42)				>30	NR	30.4*	(28.0–33.0)	30.4*	(27.1–34.0)			
	Training group (12-month. aerobic. BMI < 30)	57 (53)	58.1	5.0		<30	NR	18.6*	(16.8–20.6)	16.2*	(14.3–18.3)			
	Training group (12-month. aerobic. BMI > 30)	60 (53)				>30	NR	29.4*	(26.9–32.0)	25.6*	(23.0–28.5)			
**Beavers et al. (2013)** [33]	Control group	93	67.0	4.8	M + F (F%: NR)	32.6	3.5	37.9	22.2	36.8 (2nd phase: 40.6)	25.1 (28.9)	NR	✓	NR
	Training group (6- and 18-month. aerobic)	97				32.8	3.9	38.0	25.6	34.9 (2nd phase: 37.3)	26.6 (26.6)			
**Courteix et al. (2015)]** [34	Control group	44	57.9	47.8	M + F (F%: 47.8)	< 25	NR	12.1	10.8	NR	NR	NR	NR	✓
	Training group (3- and 12-month. moderate-resistance-moderate-endurance)	33	60.7	5.5	M + F (F%: 63.6)	< 35	NR	34.60	16.80	24.6 (2nd phase: 22.8)	15.1 (14.1)			
	Training group (3- and 12-month. high-resistance-moderate-endurance baseline)	30	62.2	4.5	M + F (F%: 60)	< 35	NR	27.40	14.40	16.6 (2nd phase: 18.1)	17.2 (13.3)			
	Training group (3- and 12-month.moderate-resistance-high-endurance baseline)	27	58.4	5.0	M + F (F%: 48.1)	< 35	NR	29.50	15.30	22.4 (2nd phase: 16.5)	24.0 (12.0)			
**Di Blasio et al. (2011)** [35]	Training group (4-month. aerobic)	22	53.1	3.1	F	28.7	3.2	35.0	16.7	24.1	14.7	✓	NR	NR
**Fatouros et al. (2005)]** [36	Control group	10	69.8	5.1	M	28.7	2.1	9.50	0.80	9.40	0.70	✓	✓	NR
	Training group 1 (6-month. light-intensity, resistance)	14	71.1	3.6		30.1	3.5	9.10	0.70	8.80	0.70			
	Training group 2 (6-month. moderate-intensity, resistance)	12	69.7	3.8		29.0	2.8	8.90	0.60	8.70	0.40			
	Training group 3 (6-month. high-intensity, resistance)	14	70.8	2.8		29.9	4.2	9.70	0.60	7.80	0.60			
**Frank et al. (2005)** [37]	Control group	86	60.6	6.8	F	30.5	3.7	26.6	(24.7–28.6)	26.3 (2nd phase: 26.5)	[23.6–29.4 (24.0–29.3)]	✓	NR	NR
	Training group (3- and 12-month. moderate-intensity. aerobic)	87	60.7	6.7		30.4	4.1	27.3	(24.6–30.2)	24.4 (2nd phase: 25.3)	[22.0–27.1 (22.8–28.1)]			
**Friedenreich et al. (2011)** [38]	Control group	160 (154)	60.6	5.7	F	29.20	4.3	19.5*	17.7–21.4	18.5 (2nd phase: 19.1)*	[16.7–20.4 (17.4–21.1)]	✓	✓	NR
	Training group (6- and 12-month. aerobic)	160 (154)	61.2	5.4		29.10	4.5	18.8*	17.3–20.4	14.8 (2nd phase: 14.9)*	[13.5–16.2 (13.5–16.5)]			
**Glynn et al. (2015)** [39]	Control group	10	50.0	9.5	M + F (F%: 50)	22.6	1.6	6.3	9.8	NR	NR	✓	✓	✓
	Training group (6-month. aerobic and resistance)	13 (9)	52.0	7.2	M + F (F%: 53.8)	30.9	2.9	31.6	26.7	23.4	23.4			
**Guelfi et al. (2013)** [40]	Control group	8	49.0	7.0	M	30.1	6.1	23.0	23.5	21.1	18.1	✓	NR	✓
	Training group 1 (12 week. resistance)	13				30.3	3.5	20.0	10.5	16.6	8.6			
	Training group 2 (12 week. aerobic)	12				31.7	3.5	19.7	7.8	15.4	7.3			
**Klimcakova et al. (2006)** [41]	Training group (12 week. strength)	12	50.4	2.3	M	33.6	3.9	16.6	6.3	13.1	5.7	✓	✓	✓
**Malin et al. (2014)** [42]	Training group (12 week. aerobic)	20	66.4	4.0	M + F (F%: NR)	34.1	4.9	20.4	13.4	15.0	13.0	✓	NR	✓
**Maltais et al. (2016)** [43]	Training group (4-month. resistance)	10	64.0	4.5	M	25.9	3.1	5.6	6.5	4.1	4.3	✓	NR	✓
**Numao et al. (2012)** [44]	Training group (12 week. aerobic)	29	48.0	10.8	M	29.60	3.8	8.50	4.80	4.70	3.80	✓	✓	✓
**O'Leary et al. (2006)** [45]	Training group (12 week. aerobic)	16	63.0	4.0	M + F (F%: 68.8)	33.2	5.6	27.0	14.4	21.4	12.3	✓	✓	✓
**Saafi et al. (2012)** [46]	Non-obese. low fitness group	11	55.0	5.0	M	25.00	2.0	3.8	1.4	NR	NR	✓	NR	NR
	Non-obese. high fitness group	36						4.1	1.9					
	Obese. low fitness group	72	57.0	6.0		30.00	4.0	11.5	6.7					
	Obese. high fitness group	50						7.9	3.8					
**Shah et al. (2011)** [47]	Control group	27	69.0	4.0	NR	37.3	4.7	33.2	17.7	30.1 (2nd phase: 33.9)	18.4 (20.5)	✓	NR	NR
	Training group (6- and 12-month aerobic and resistance)	26	70.0	4.0		36.9	5.4	34.4	21.9	31.7 (2nd phase: 33.9)	22.6 (24.1)			
**Sjögren et al. (2012)** [48]	Control group	43	67.5	0.5	M + F (F%: 41.9)	30.30	0.8	22**	5.8	20.9**	7.1	✓	✓	✓
	Training group (6-month. aerobic and resistance)	30	67.6	0.5	M + F (F%: 63.3)	27.50	1.1	15**	2.5	13.2**	4.8			
**Solomon et al. (2008)** [49]	Training group (12 week. aerobic)	12	66.0	3.5	M + F (F%: NR)	34.7	5.5	25.8	13.5	22.4	12.1	✓	✓	✓
**Tan et al. (2016)** [50]	Control group	11	49.7	7.9	F	27.80	1.5	11.9	6.3	12.3	5.6	✓	NR	✓
	Training group (10 week. aerobic)	11	50.7	5.5		28.50	2.1	11.3	5.5	8.2	1.1			

F, female; M, male; NR, not reported;

*, geometric mean;

**, calculated from median and corresponding interquartile values (IQR) [31]

**Table 2:** Training-induced improvement in parameters of carbohydrate metabolism.

Study	Subgroup	Baseline metabolic state	Glucose		Insulin	
Fatouros et al. 2005 (36)	Training group 1 (6-month, light-intensity, resistance)	non-diabetic	fasting	*	HOMA	*
	Training group 2 (6-month, moderate-intensity, resistance)	non-diabetic	fasting	*	HOMA	*
	Training group 3 (6-month, high-intensity, resistance)	non-diabetic	fasting	*	HOMA	*
Frank et al. 2005 (37)	Training group (3-month, moderate-intensity, aerobic)	non-diabetic	NR		HOMA	*
Friedenreich et al. 2011 (38)	Training group (6-month, aerobic)	healthy	fasting	NS	fasting	*
					HOMA	*
Glynn et al. 2015 (39)	Training group (6-month, aerobic and resistance)	insulin resistant	fasting	NS	fasting	*
			2 h, OGTT	*	HOMA	*
Guelfi et al. 2013 (40)	Training group 1 (12 week, resistance)	NR	postprandial, over 2 h	NS	postprandial, over 2 h	NS
	Training group 2 (12 week, aerobic)	NR	postprandial, over 2 h	*	postprandial, over 2 h	*
Klimcakova et al. 2006 (41)	Training group (12 week, strength)	mixed (diabetic, prediabetic, healthy)	fasting	NS	fasting	NS
			disposal rate	*	euglycemic-hyperinsulinemic clamp	*
Malin et al. 2014 (42)	Training group (12 week, aerobic)	non-diabetic	fasting	*	fasting	*
			2 h, OGTT	*	2 h, OGTT	*
Maltais et al. 2016 (43)	Training group (4-month, resistance)	NR	fasting	NS	fasting	NS
Numao et al. 2012 (44)	Training group (12 week, aerobic)	non-diabetic	fasting	NS	fasting	NS
			HbA1c	*	QUICKI	*
O'Leary et al. 2005 (45)	Training group (12 week, aerobic)	non-diabetic	fasting	NS	fasting	*
			OGTT, AUC	*	OGTT, AUC	*
Sjögren et al. 2012 (48)	Training group (6-month, aerobic and resistance)	healthy	fasting	NS	fasting	*
			HbA1c	NS	HOMA	NS
Solomon et al. 2008 (49)	Training group (12 week, aerobic)	prediabetic (IGT)	NR		euglycemic-hyperinsulinemic clamp	*
Tan et al. 2014 (50)	Training group (10 week, aerobic)	non-diabetic	fasting	*	NR	

AUC, area under the curve; HbA1c, hemoglobin A1c; HOMA, homeostatic model assessment of insulin resistance; IGT, impaired glucose tolerance; NR, not reported; NS, non-significant; OGTT, oral glucose tolerance test; QUICKI, quantitative insulin sensitivity check index

*, significant (p < 0.05) difference between pre- and first post training values

Regarding gender, 6 studies reported data of men [36, 40, 41, 43, 44, 46], 5 reported data of women [32, 35, 37, 38, 50], the remaining 8 merged data of men and women [33, 34, 39, 42, 45, 47–49].

Mean age value (with SD) was given for a wide age-range of the participants. It exceeded 45 years in all 19 studies [32–50], while 6 of them also reported data of participants with mean age above 65 years [33, 36, 42, 47, 48, 49].

Overall, the quality of the evidence base was downgraded to low, based on the GRADE system according to the BMJ Clinical Evidence approach [30] (S3 Table).

### Primary analysis and publication bias

#### Effect of training on BMI

The weight loss proved to be significant in 9 groups [36, 40, 42, 44, 45, 49, 50]. Meta-analysis of differences in post-training vs. pre-training BMI values confirmed a significant suppressing effect of training (not shown). Weighted overall effect size (ES) was -0.71 with 95% CI of -1.37, -0.05, p = 0.034. Heterogeneity of the data was low: I^2^ = 0.00%, p = 1.0. No publication bias was indicated by Egger’s test: p = 0.715.

#### Effect of training on blood leptin level

**Fig 2:**
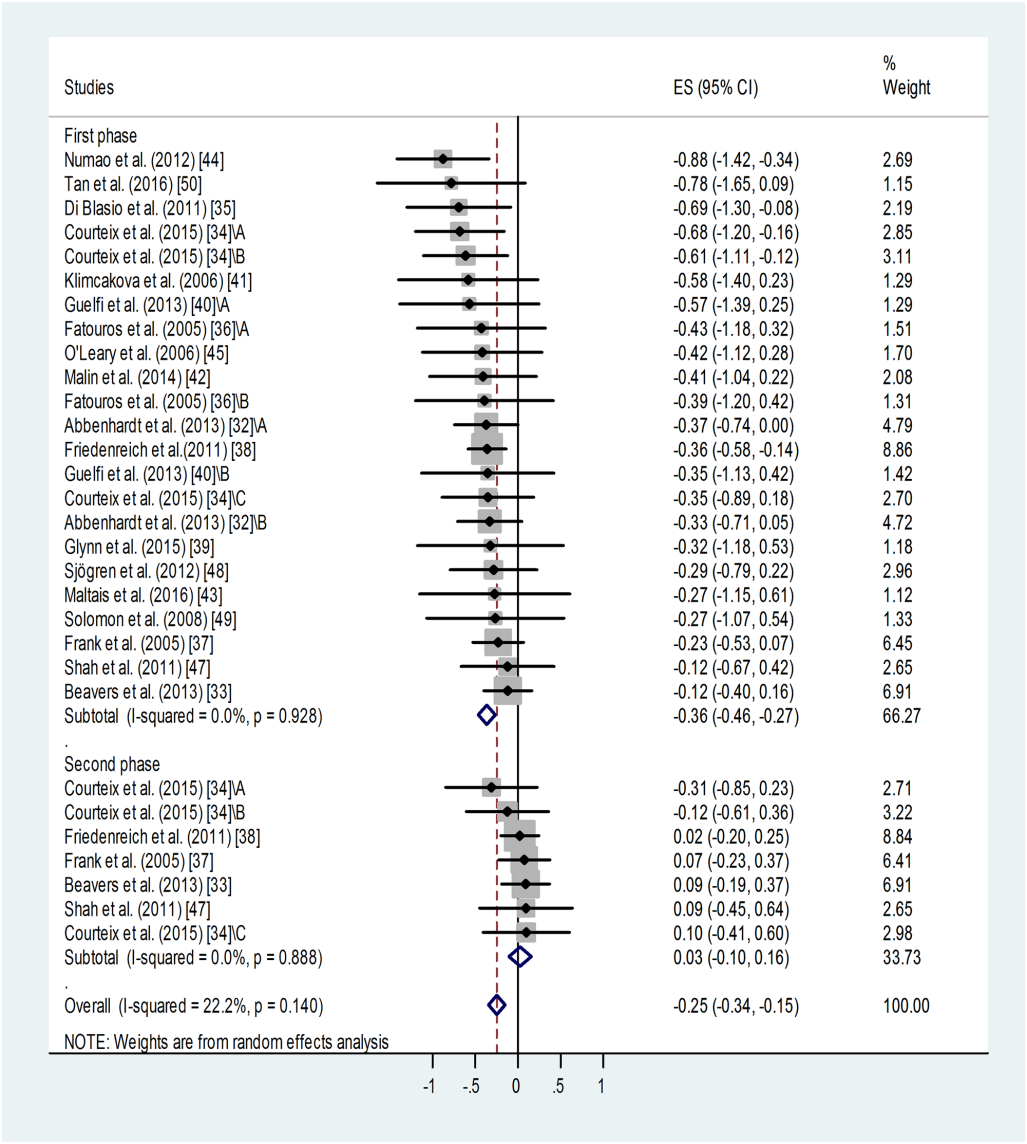
Forest plot representing standardized differences between the mean post- and pre-training blood leptin values of participants in the first phase and second phase (when available) of a training intervention. When more than one group (of varying ages or BMI categories) participated in physical training (of varying training types or intensities) within the same study, letters A, B or C indicated them. Regarding second phase values, standardized differences were calculated from values measured at the end of the first and second phases (via subtraction of post-first phase value from post-second phase value). Squares show the standardized difference in mean values with the grey area reflecting the weight assigned to the study. Horizontal bars indicate 95% confidence intervals (95% CI). The diamond shows the overall effect size (ES) with its corresponding 95% CI.

Meta-analysis of standardized differences in post-training *vs*. pre-training blood leptin levels reported by 18 studies for 23 training groups [32–45, 47–50] confirmed a significant suppressing effect of training for the first or only training period (Fig 2). Weighted overall effect size (ES) was -0.36 with 95% CI of -0.46, -0.27, p < 0.001. Heterogeneity of the data was low: I^2^ = 0.00%, p = 0.928. Publication bias was indicated by Egger’s test: p = 0.048, i.e. an asymmetry of the funnel plot. This may be (at least partly) attributed to “small-study effect”. Studies with relatively big standard deviations tend to appear on the left side of the funnel plot (S1 Fig) causing its asymmetry. Results of small studies with big standard deviations have a better chance to be published if they report positive, significant outcomes.

Based on the Duval and Tweedie's trim and fill algorithm, 8 additional studies were needed to balance the asymmetry. This method yielded the adjusted value of -0.289 (95% CI: -0.38, -0.20). When we compare the adjusted value (-0.289) with the original one (-0.36), it implies that the small study effect did contribute to our original results. However, without this publication bias, the fill and trim algorithm still showed a significant and relatively large effect.

Regarding those studies in which two post-training measurements were available [11, 12, 14, 19, 24], continued training failed to induce further decrease in the leptin level or it was rather associated with some relapse (Fig 2, Table 1). Weighted overall ES was 0.03 with 95% CI of -0.10, 0.16, p < 0.001. Heterogeneity of the data was low: I^2^ = 00.0%, p = 0.888. No publication bias was identified using Egger’s test: p = 0.35 for this subgroup.

#### Effect of BMI category on training-induced suppression of blood leptin level

**Fig 3:**
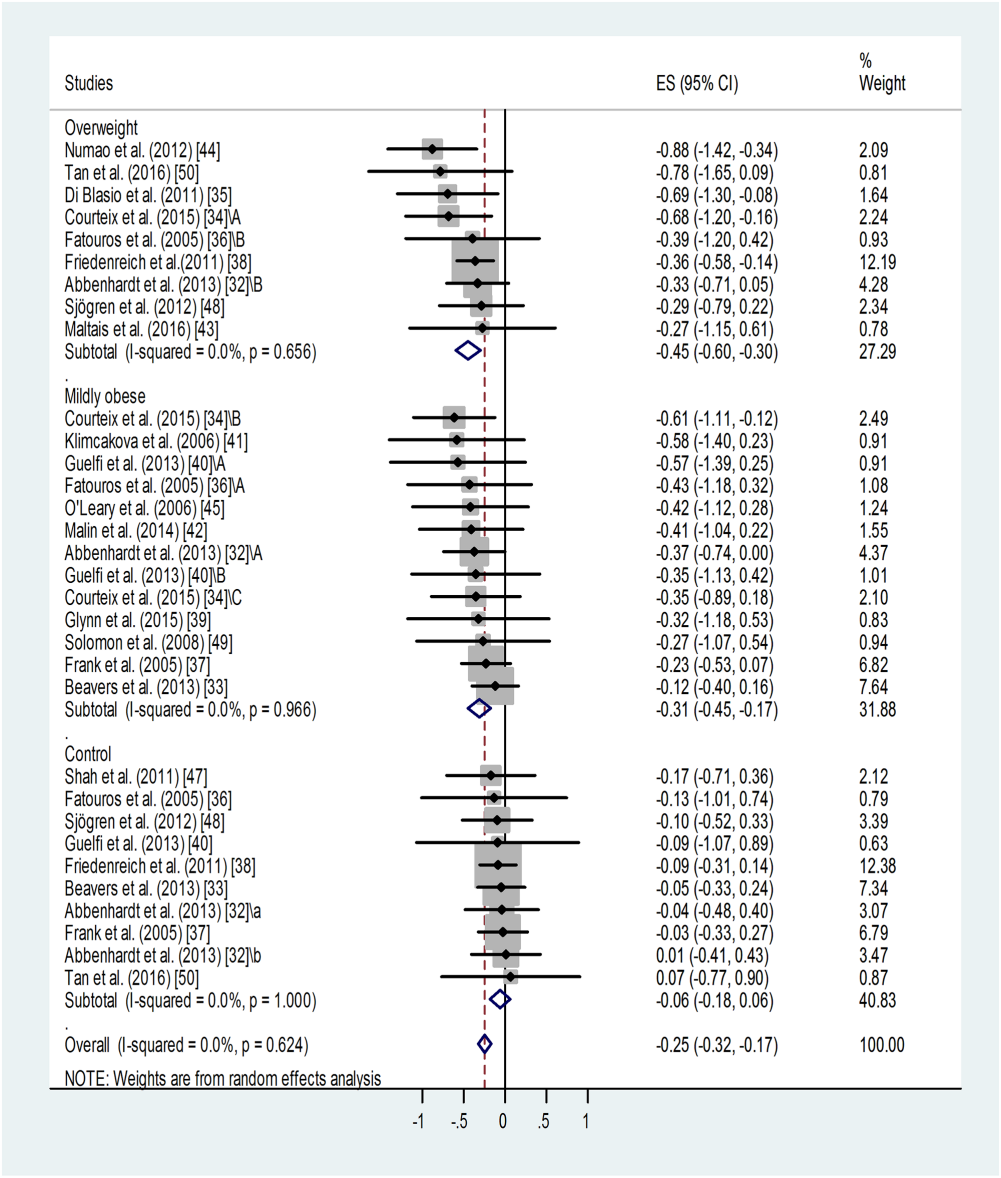
Forest plot representing standardized differences between the mean post- and pre-training blood leptin values of overweight and mildly obese participants in the first phase of a training intervention. When more than one group participated in physical training within the same study, letters A, B or C indicated them.Variations of leptin levels of sedentary controls are also shown. Squares indicate the standardized difference in mean values with the grey area reflecting the weight assigned to the study. Horizontal bars represent 95% confidence intervals (95% CI). The diamond shows the overall effect size (ES) with its corresponding 95% CI.

A similar meta-analysis of sub-groups of different BMI categories showed that training led to similar, significant suppressions of leptin level in the overweight (p < 0.001) and in the mildly obese groups (p < 0.001) (Fig 3). Only one study reported results for a severely obese training group without detecting any effect [47]. Training effects in the overweight and mildly obese groups were different from results obtained in sedentary controls. For non-trained controls: weighted overall ES = -0.06 with 95% CI (-0.18, 0.06). For overweight training groups: weighted overall ES = -0.45 with 95% CI (-0.60, -0.30). For mildly obese training groups: weighted overall ES = -0.31 with 95% CI (-0.45, -0.17).

Overall heterogeneity of the data was low in all cases: I^2^ = 0.00% (for the overweight group p = 0.656, for the mildly obese group p = 0.966, for controls p = 0.624).

Some publication bias has been detected by the Egger’s test: p < 0.1 for the overweight and p < 0.001 for the mildly obese groups. Two additional studies were needed to balance the asymmetry in case of the overweight and 6 for the mildly obese groups (not shown). Adjusted values based on the Duval and Tweedie's trim and fill algorithm were ES = -0.40 with 95% CI (-0.54, -0.26) for the overweight and ES = -0.24 with 95% CI (-0.36, -0.12) for the mildly obese group. The fill and trim algorithm still showed significant effects in both cases.

#### Effect of gender on training-induced suppression of blood leptin level

**Fig 4:**
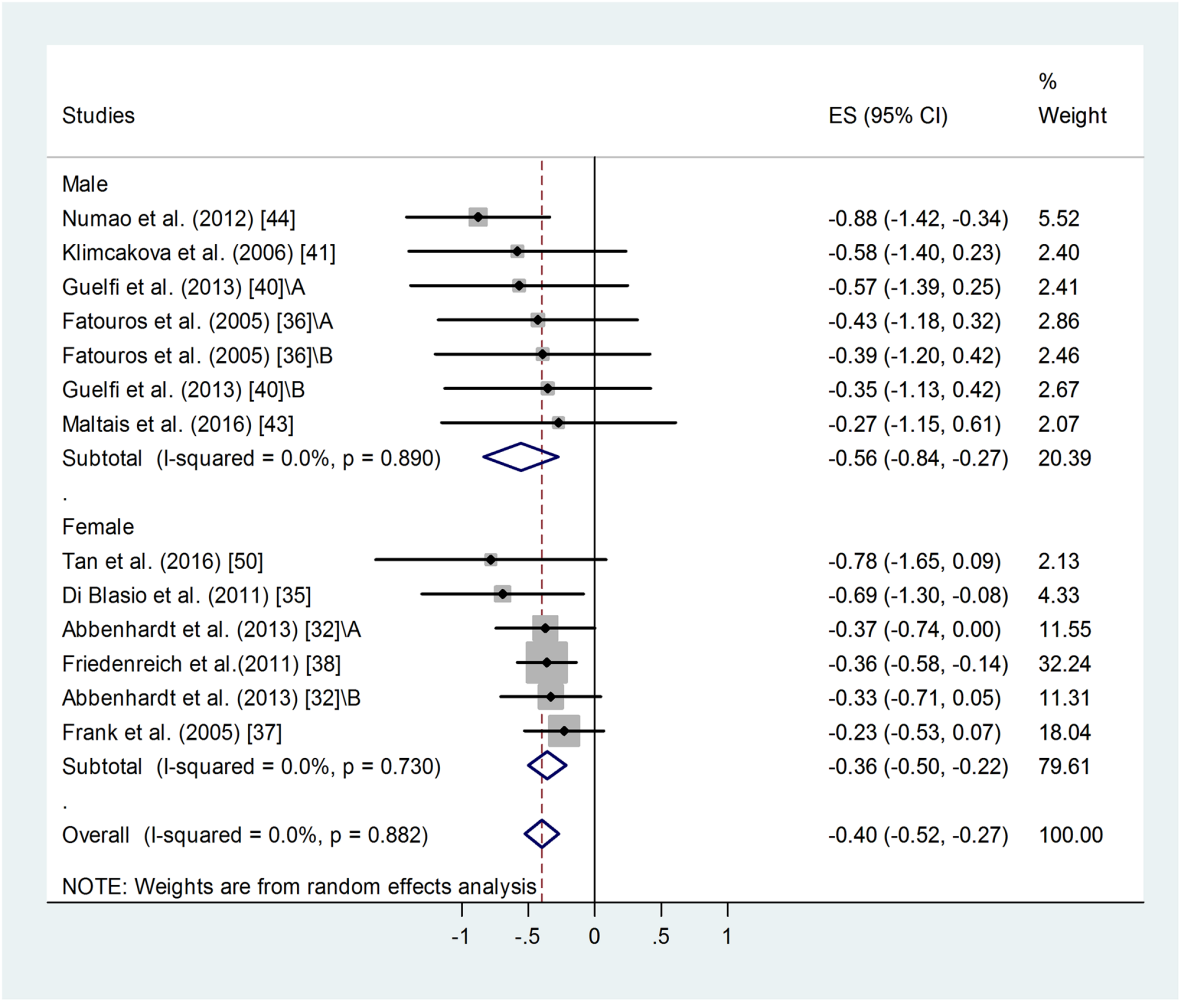
Forest plot representing standardized differences between the mean post- and pre-training blood leptin values of men and women in the first phase of a training intervention. When more than one group participated in physical training within the same study, letters A or B indicated them. Squares indicate the standardized difference in mean values with the grey area reflecting the weight assigned to the study. Horizontal bars represent 95% confidence intervals (95% CI). The diamond shows the overall effect size (ES) with its corresponding 95% CI.

A similar meta-analysis of male and female sub-groups showed a somewhat stronger training effect in men: weighted overall ES = -0.56 with 95% CI (-0.84, -0.27), p < 0.001 (Fig 4). For female training groups: weighted overall ES = -0.36 with 95% CI (-0.50, -0.22), p < 0.001. There was no difference between the two groups.

Overall heterogeneity of the data was low: I^2^ = 0.00%, for men p = 0.890, for women p = 0.730. Similar publication bias was identified in male and female groups using Egger’s test: p < 0.1 in both cases. Two additional studies were needed to balance the asymmetry in case of female groups and 3 for the male ones. Adjusted values based on the Duval and Tweedie's trim and fill algorithm were ES = -0.33 with 95% CI (-0.46, -0.20) for the female and ES = -0.67 with 95% CI (-0.91, -0.43) for the male participants. The fill and trim algorithm still showed a significant effects in both cases.

#### Effect of age on training-induced suppression of blood leptin level

**Fig 5:**
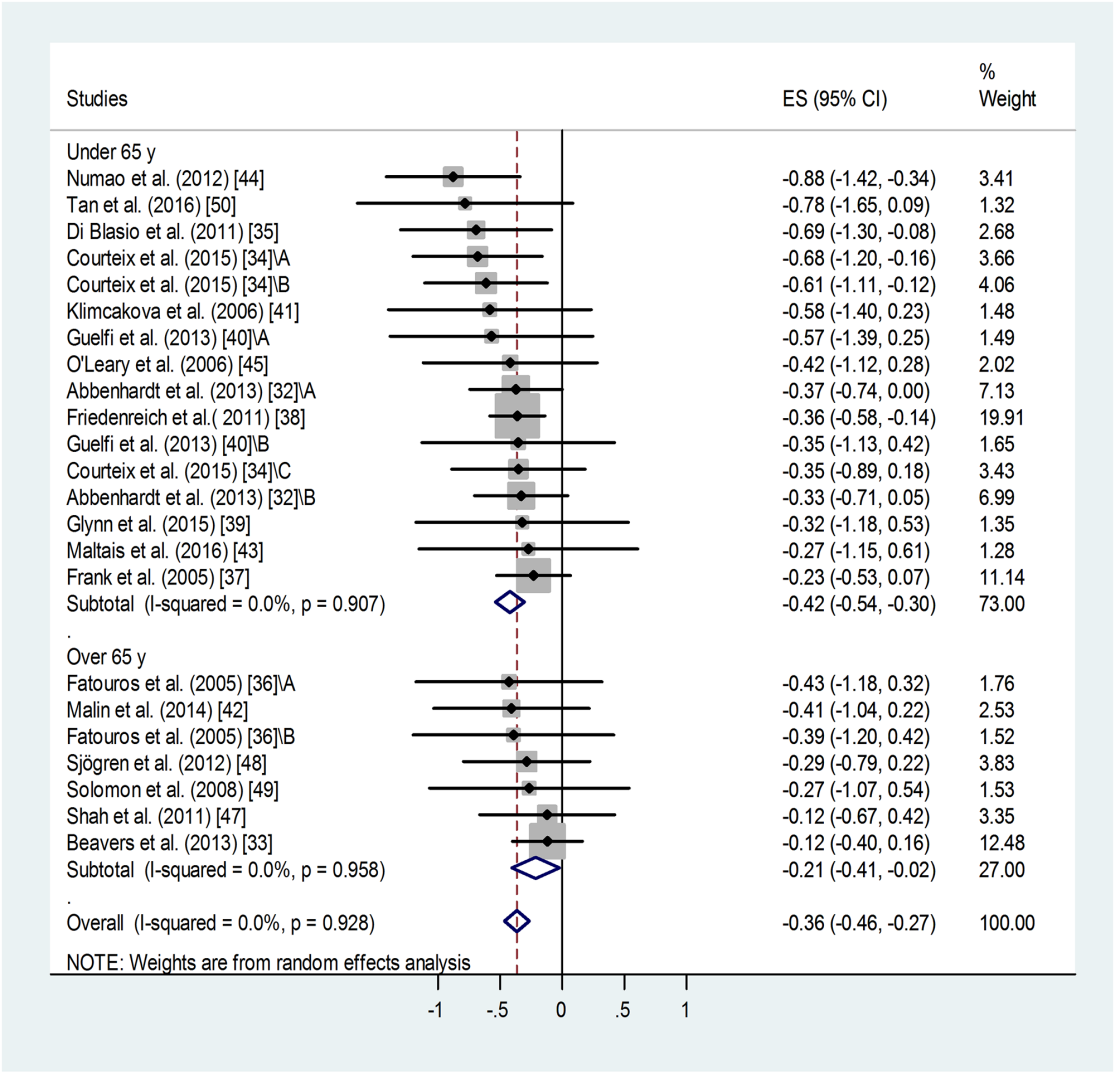
Forest plot representing standardized differences between the mean post- and pre-training blood leptin values of middle-aged (45 years < mean age < 65 years) and older (mean age ≥ 65 years) age-groups in the first phase of a training intervention. When more than one group participated in physical within the same study, letters A, B or C indicated them. Squares indicate the standardized difference in mean values with the grey area reflecting the weight assigned to the study. Horizontal bars represent 95% confidence intervals (95% CI). The diamond shows the overall effect size (ES) with its corresponding 95% CI.

Meta-analysis of standardized differences in post-training *vs*. pre-training blood leptin levels of middle-aged (45 years < mean age < 65 years) and older sub-groups (mean age ≥ 65 years) found similar, significant effects in both groups (Fig 5). For the younger training groups: weighted overall ES = -0.42 with 95% CI (-0.54, -0.30), p<0.001. (Fig 5). For the older training groups: standard difference of means = -0.36 with 95% CI (-0.46, -0.27), p = 0.029. There was no significant difference between the two groups, although the younger group showed a tendency to benefit more from the training.

Heterogeneity of the data was low: I^2^ = 00.00%, for the middle-aged group p = 0.907, for the older group p = 0.958. Similar publication bias was identified for both age-groups: p < 0.1 in both cases. Four additional studies were needed to balance the asymmetry in case of the middle-aged and 3 for the older age-group. Adjusted values based on the Duval and Tweedie's trim and fill algorithm were ES = -0.36 with 95% CI (-0.47, -0.25) for the middle-aged and ES = -0.16 with 95% CI (-0.33, +0.01) for the older age-group. The fill and trim algorithm still showed significant effects in both cases.

#### Effect of training on blood adiponectin level

Ten studies reported additional pre- and post-training adiponectin levels [32, 33, 36, 38, 39, 41, 44, 45, 48, 49]. Results of the only or first post-training adiponectin measurements were used, because insufficient data was available for the 2^nd^ training periods (only two studies reported such data). Meta-analysis of standardized differences in post-training *vs*. pre-training blood adiponectin levels failed to demonstrate any effect of training (not shown). Weighted overall ES = 0.008 with 95% CI of -0.116, 0.132, p = 0.646. Heterogeneity of the data was low: I^2^ = 0.00%, p = 0.747. No publication bias was identified: p = 0.156.

### Additional analyses

#### Effect of fitness category on blood leptin level

Weighted mean peripheral leptin values of subgroups of all nineteen studies divided by fitness categories (from sedentary to very active, from 4 to 1, respectively) were also analysed (not shown).

For category 4: overall weighted mean was 26.08 ng/ml, 95% CI (23.80, 28.36). For category 3: overall weighted mean was 21.26 ng/ml, 95% CI (17.73, 24.79). For category 2: overall weighted mean was 17.39 ng/ml, 95% CI (14.14, 20.64). For category 1: overall weighted mean was 6.72 ng/ml, 95% CI (3.84, 09.60). Difference was detected between the very active (1) and sedentary groups (2–4, p< 0.001) and also between categories 2 and 4.

High value of heterogeneity (I^2^: between 94.97 and 97.78% in all categories, p < 0.001) indicates the contribution of other strong factors determining these values.

Significant publication bias was detected, p < 0.05 in all categories. As shown before (S1 Fig), such a publication bias had a relatively weak effect on our results.

#### Effect of training type on correlation between fat loss and change in leptin level

**Fig 6:**
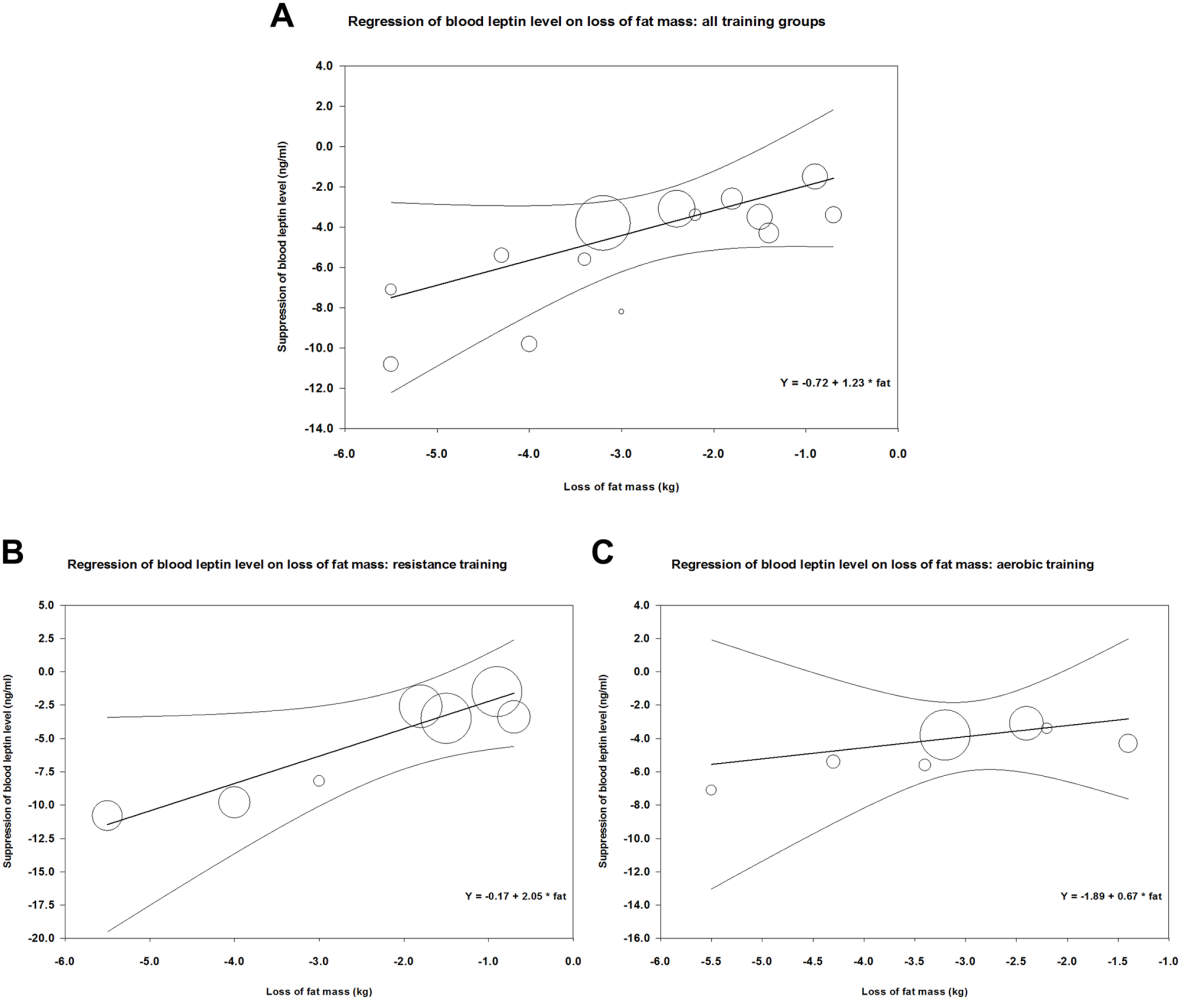
Meta-regression of training-induced suppression of blood leptin levels *versus* loss of fat mass using data from our analyzed studies (Table 1). Panel A shows the correlation between loss of fat and suppression of leptin level for all data; Panel B demonstrates a similar correlation for resistance or mixed training, while Panel C shows the correlation for aerobic training.

Fig 6 demonstrates the correlation between loss of fat mass and decrease in mean blood leptin level during training in 14 study groups of those of our analyzed studies that also reported changes of fat mass [34, 39–45, 48–50] (parameters of meta-regression between loss of fat mass and suppression in leptin levels were: number of groups: 14, coefficient: 1.23, p = 0.048). Concerning the effects of aerobic *vs*. resistance or strength training (or mixed) on the correlation between reduction in fat mass and suppression of leptin level, meta-regression showed a steeper decline in case of resistance than in case of aerobic training (Fig 6 panels B and C). In case of resistance training, parameters of meta-regression were: number of groups: 7, coefficient: 2.05, p = 0.0167, whereas in case of aerobic training: number of groups: 7, coefficient: 0.67, p = 0.562.

In all cases, the goodness of fit test shows that the unexplained variance is zero, thus it does not indicate the presence of other determining factors (for the resistance groups: Q = 0.66, df = 5, p = 0.99, for the aerobic groups: Q = 0.52, df = 5 p = 0.99, for the overall analysis Q = 3.99, df = 12, p = 0.99).

#### Effect of training on parameters of carbohydrate metabolism and insulin level

Training also led to some improvement in parameters of carbohydrate metabolism. Insulin levels were mainly affected, glucose levels improved to a lesser extent [36–45, 48–50] (Table 2). Although improvement of parameters of carbohydrate metabolism was maintained until the end of the second phase of training (when present) [37, 38], no further improvement was observed.

## Discussion

Lifestyle interventions aiming at weight loss in overweight or obese adult populations have been shown to reduce mortality in all age-groups, regardless of successful achievement of weight loss [51]. Previous studies have demonstrated that chronic endurance exercise that increases muscle strength suppresses blood leptin levels [52, 36, 53], but no clear correlation between muscle strength or fitness and suppression of leptin level has been established [54]. Some authors argue that the effects of physical training on leptin levels may not be independent of consequent weight loss [55].

Our present study aimed to review the literature and meta-analyze previous findings on the effects of physical training intervention *per se* (without dietary intervention or major weight loss) on peripheral leptin level as indicator of metabolic health in middle-aged or older overweight or obese populations. Our objective was to evaluate the independent efficacy of this intervention type concerning peripheral leptin levels and to suggest new approaches for future clinical studies.

Our results show that physical training leads to a decrease in leptin level of middle-aged or older (above 45 years of age) overweight or obese people. These training interventions also resulted in a slight but significant suppressing effect on BMI (average decrease in BMI < 2.5%). Therefore, we cannot conclude that the beneficial effects of training intervention on blood leptin level is independent of the weight-reducing effects. The link between weight loss and amelioration of blood leptin is also reinforced by our findings that confirm close correlation between training-induced fat loss and change in leptin levels (Fig 6) [56–58]. In those clinical studies of our analyses where both types of data were available [34, 39–45, 48–50], training-induced suppression of leptin did indeed show close correlation with loss of fat mass. (The regression line was steeper in case of resistance training interventions than in case of aerobic ones.) However, we would like to emphasize that this weight loss was very moderate (amounting to a 2–3 kg weight loss in obese individuals in the BMI range of 30–40). In addition, the lack of increase in the serum adiponectin level in our analyzed studies could appear as an indication of a moderate weight loss, as serum adiponectin levels were reported to increase in interventions that induced major weight loss or significant reduction of visceral fat [56, 48, 57, 58]. However, adiponectin is a complex biomarker, so the lack of effect of training on serum adiponectin could be also attributed to other factors independent of body fat (e.g. insulin sensitivity or lipid profile) [59]. Regarding further potential mechanisms of the training-induced reduction of blood leptin the potential reduction of the ratio of visceral fat (unfortunatelly not reported in the analyzed studies) or the training-induced reduction of intramyocellular lipid content suggested by previous observations [4, 7] should also be taken into consideration.

Within the age range from 45 to 80, mean age values of participant groups did not appear to influence the beneficial effects of physical training on leptin levels. This finding is especially important, because hyperleptinemia has been suggested to promote the development of age-associated cardiometabolic abnormalities, such as hypertension, atherosclerosis or microcirculatory disorders [13, 22, 60] or that of autoimmune diseases, e.g. rheumatoid arthritis or systemic lupus erythematosus [61]. Therefore, it seems that older age-groups may also benefit from physical training with regard to blood leptin level.

Our results also showed that both overweight and mildly obese participants drew significant benefit of physical training, regarding suppression of blood leptin level. Training effects could not be assessed in severely obese groups, since only one study was conducted in those patients [47]. It would be an important future objective to conduct clinical interventions in such high-risk groups, as well.

Both men and women benefited from the training intervention, although the mean effects seemed somewhat bigger in men. This tendency is in accord with previous findings [62]. These findings are especially interesting, because men, in general, have lower leptin levels than women [62]. Higher muscle mass of male participants may explain this difference in baseline leptin levels and in training efficacy. This assumption is further supported by our present findings, according to which resistance or strength training that promotes increase in muscle mass led to a higher rate of suppression of leptin for the same rate of fat loss than aerobic training. In addition, weighted means of blood leptin levels showed higher values in sedentary than in more fit categories. Concerning the potential mechanisms of these findings, previous observations demonstrated that training leading to bigger muscle strength and lower fatty infiltration of muscles were associated with increased expression of leptin receptors that can bind more leptin. Increased muscle mass also contributes to increased fat utilisation leading to a reduction of fat mass. This reduction in fat mass has also been hypothesized to increase circulating soluble leptin receptor level and consequently to reduce serum leptin [54].

Parallel with suppression of leptin level, training also led to some improvement in parameters of carbohydrate metabolism. These improvements were maintained until the end of the second phase of training, unlike improvement in leptin level. These results are in accord with those previous findings suggesting a strong effect of training on insulin resistance [63, 64, 45]. Some studies suggested that exercise itself is sufficient to improve carbohydrate metabolism [63], while others emphasized the role of visceral fat loss [38], or at least a moderate rate (reaching at least 3%) of weight loss [64, 45]. Still other researchers argue that only a major (at least 10%) reduction in BMI [59] or visceral fat [65] can lead to such an improvement.

### Limitations

Some limitations of our meta-analysis stem from inconsistencies of the available studies. Initial fitness status of participants was usually poorly defined. In this regard, studies lack standardized data such as muscle strength (preferably lower body), muscle mass or maximal oxygen consumption. Training protocols were also widely different concerning overall duration, methods, intensity, placement of evaluation points. Lack of special data concerning visceral versus subcutaneous fat limited the value of corresponding analysis. Lack of standardized data for the evaluation of carbohydrate metabolism and insulin resistance also impeded evaluation of the metabolic benefits of training. Although our meta-analysis focused on benefits of training and not on dietary interventions, it could have improved the reliability of results if caloric consumption and dietary habits of the participants (e.g. the ratio of saturated and polyunsaturated omega-3 fatty acids, antioxidants, etc.) were also assessed. It is possible that caloric content and macro- and micronutrient composition of individual diets could also influence study outcomes [39].

Limitations of our study also include the presence of publication bias regarding the training-induced suppression of mean blood leptin level. Small-study effect probably contributed to the appearance of this bias. However, without this publication bias, the fill and trim algorithm still showed a significant and relatively large effect of training on blood leptin level (S1 Fig).

The risk of bias may be further increased by the lack of prior registration of the protocol of our meta-analysis.

In the available studies, mean or median age value was given for a wide age-range of the participants limiting the strength of analysis of age as an influencing factor. Regarding leptin and adiponectin values, some papers presented their data as median and quartiles or geometrical means. We were able to convert median values (with quartiles) into mean ± standard deviation using the transformation suggested by Wan et al. [31], but no conversion is available for geometrical means. For this reason, we applied standardized difference of means in our primary analyses concerning leptin or adiponectin levels. Some additional limitations are also derived from shortcomings of our meta-regression analyses. Regression of changes in leptin levels and fat loss was calculated for different training types, although fat loss values were only available as means with standard deviations. The inclusion of two pre-diabetic subgroups and a mixed one of healthy, prediabetic and a few diabetic patients may have reduced our estimate of training effects. Controversial data concerning blood leptin levels of type 2 diabetic patients *versus* non-diabetic obese groups have been reported [66–69]. Some studies suggested that diabetic patients have higher leptin levels that are more difficult to suppress [66, 67], others describe lower leptin levels in prediabetic or diabetic patients [68, 69].

## Conclusions

Our meta-analysis suggests the potential contribution of training intervention to the improvement of metabolic status as indicated by lower leptin levels in middle-aged or older overweight or mildly obese populations, especially concerning resistance or mixed (strength and aerobic) training types. In the future, further randomized longitudinal studies are needed to properly evaluate the effect of different types (resistance, aerobic or combined), intensities and durations of physical training on standardized parameters of carbohydrate metabolism, leptin and adiponectin levels. These studies should also test very obese middle-aged and elderly populations, as well, since hardly any data are available concerning such high-risk groups.

## Supporting information

S1 Table PRISMA checklist of the meta-analysis. (PDF)

S2 Table Assessment of risk of bias of individual studies. Risk of bias assessment was based on the Cochrane Collaboration's tool [29].
(PDF)

S3 Table Evaluation of risk of bias across all the studies of the meta-analysis. Regarding the quality of the evidence base as a whole, the scoring system used for Clinical Evidence reviews, GRADE was applied [30].
(PDF)

S1 Fig Funnel plot with Duval and Tweedie's trim and fill algorithm. Panel A shows the asymmetrical funnel plot representing the publication bias regarding training-induced suppression of blood leptin values (first or only phase of training). Panel B shows the results of the Duval and Tweedie's trim and fill algorithm method. The 8 additional studies that were needed to balance the asymmetry of the funnel plot are indicated by full black symbols. Original results (23 data points) are represented by empty ones. The empty diamond indicates the original effect size, while the full, black one shows the adjusted effect size.
(TIF)
