# Supplementary material for: In middle-aged and old obese patients, training intervention reduces leptin level: A meta-analysis
Source: PLoS One. 2017 Aug 15;12(8):e0182801. doi: 10.1371/journal.pone.0182801 (PMC5557366; doi:10.1371/journal.pone.0182801)
Supplement: S2 Table — Risk of bias assessment was based on the Cochrane Collaboration's tool [29]. (PDF) [file pone.0182801.s002.pdf]

S2 Table. Assessment of risk of bias of individual studies

Risk of bias assessment was based on the Cochrane Collaboration's tool [29].

Study, reference	Random sequence generation	Allocation concealment	Blinding of personnel	Blinding of outcome assessment	Incomplete outcome data	Selective reporting	Overall risk of bias
**Abbenhardt et al.[32]**	Yes	No	Yes	Unclear	Low risk	Low risk	Low risk
**Beavers et al. [33]**	Yes	No	Yes	Unclear	Low risk	Low risk	Low risk
**Courteix et al.[34]**	Yes	No	Yes	Unclear	Low risk	Low risk	Low risk
**Di Blasio et al.[35]**	No	No	Unclear	Unclear	Low risk	Low risk	High risk
**Fatouros et al.[36]**	Yes	No	Yes	Unclear	Low risk	Low risk	Low risk
**Frank et al.[37]**	Yes	No	Yes	Unclear	Low risk	Low risk	Low risk
**Friedenreich et al. [38]**	Yes	No	Yes	Unclear	Low risk	Low risk	Low risk
**Glynn et al. [39]**	No	No	Yes	Unclear	Low risk	Low risk	Low risk
**Guelfi et al. [40]**	No	No	Yes	Unclear	Low risk	Low risk	Low risk
**Klimcakova et al. [41]**	No	No	No	Unclear	Low risk	Low risk	High risk
**Malin et al.[42]**	No	No	No	Unclear	Low risk	Low risk	High risk
**Maltais et al. [43]**	No	No	No	Unclear	Low risk	Low risk	High risk
**Numao et al. [44]**	No	No	No	Unclear	Low risk	Low risk	High risk
**O'Leary et al. [45]**	No	No	No	Unclear	Low risk	Low risk	High risk
**Saafi et al.[46]**	No	No	Unclear	Unclear	Low risk	Low risk	High risk
**Shah et al. [47]**	Yes	No	Yes	Unclear	Low risk	Low risk	Low risk
**Sjögren et al. [48]**	Yes	No	Yes	Unclear	Low risk	Low risk	Low risk
**Solomon et al. [49]**	No	No	No	Unclear	Low risk	Low risk	High risk
**Tan et al. [50]**	Yes	No	Yes	Unclear	Low risk	Low risk	Low risk
