# Supplementary material for: In middle-aged and old obese patients, training intervention reduces leptin level: A meta-analysis
Source: PLoS One. 2017 Aug 15;12(8):e0182801. doi: 10.1371/journal.pone.0182801 (PMC5557366; doi:10.1371/journal.pone.0182801)
Supplement: S3 Table — Regarding the quality of the evidence base as a whole, the scoring system used for Clinical Evidence reviews, GRADE was applied [30]. (PDF) [file pone.0182801.s003.pdf]

S3 Table. Evaluation of risk of bias across all the studies of the meta-analysis

Regarding the quality of the evidence base as a whole, the scoring system used for Clinical Evidence reviews, GRADE was applied [30].

	**Type of evidence**	**Quality** blinding and allocation follow-up and withdrawals sparse data	**Consistency** consistency of outcomes	**Directness** generalisability of population	**Effect size**	**GRADE Score** overall risk of bias
Score	+4	-1	-1	0	0	2
**Overall evaluation**	9/19 randomized clinical trials (RCT) 9/19 intervention studies	Follow up was carried out and withdrawals were reported in all studies. However, blinding was not possible in a training intervention. Blinding of personnel was reported only in 11/19 studies.	Although most studies showed similar results, there was some heterogeneity across the outcomes.	The population and the outcomes of each study show good generalisability for our population of interest.	Standardized effect sizes did not exceed 2.	Overall, the quality of the evidence base was downgraded to low. Risk of bias is assessed as moderate.
