# Supplementary figures and images for: In middle-aged and old obese patients, training intervention reduces leptin level: A meta-analysis
Source: PLoS One. 2017 Aug 15;12(8):e0182801. doi: 10.1371/journal.pone.0182801 (PMC5557366; doi:10.1371/journal.pone.0182801)

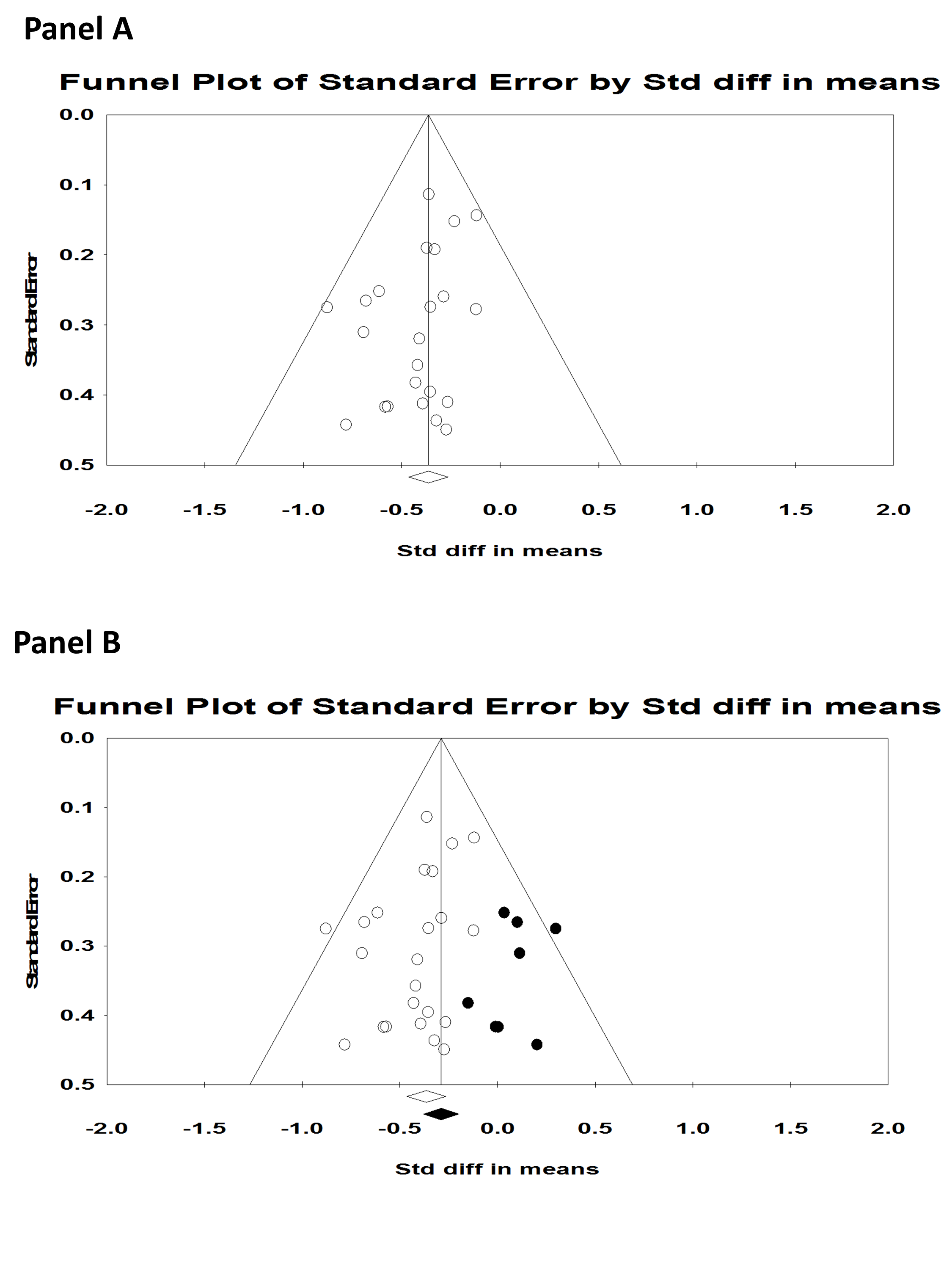

Supplement: S1 Fig — Panel A shows the asymmetrical funnel plot representing the publication bias regarding training-induced suppression of blood leptin values (first or only phase of training). Panel B shows the results of the Duval and Tweedie's trim and fill algorithm method. The 8 additional studies that were needed to balance the asymmetry of the funnel plot are indicated by full black symbols. Original results (23 data points) are represented by empty ones. The empty diamond indicates the original effect size, while the full, black one shows the adjusted effect size. (TIF) [file pone.0182801.s004.tif]
